# Supplementary material for: Monkeypox: A Histopathological and Transmission Electron Microscopy Study
Source: Microorganisms. 2023 Jul 9;11(7):1781. doi: 10.3390/microorganisms11071781 (PMC10385849; doi:10.3390/microorganisms11071781)
Supplement: Supplementary file 1 [file microorganisms-11-01781-s001.zip › microorganisms-2451630-supplementary.pdf]

Pat ID	Sex	Age	Ethnicity	Sexual behavior	Unprotected sex	Travel history	Other STIs	Kind of lesions	Number of skin lesions	Localization	Systemic symptoms	Lymphadenopathy	Hospitalization	Outcome
1	M	45	Ukrainian	MSM	Yes	No	None	Pustular lesions	>10	Genital region; lower limb; middle finger of the right hand	Fever; headache; asthenia	No	Yes	CR
2	M	34	Caucasian (Italy)	MSM	Yes	Yes (Netherlands)	None	Pustular lesions	>5	Penile shaft and glans	Fever; headache; myalgia; arthralgia; asthenia	No	No	CR
3	M	42	Caucasian (Italy)	MSM	Yes	Yes (Spain)	None	Pustular lesions	<5	Penile shaft and glans; pubic region; perioral region	Asthenia	Yes (laterocervical area)	No	CR
4	M	37	Caucasian (Italy)	MSM	Yes	No	Late-latent syphilis	Pseudo-pustular lesions	>10	Face; penile shaft; perianal region	Hyperpyrexia; rectal pain	No	No	CR
5	M	40	Brazilian	MSM	Yes	No	None	Vesico-pustular lesions	>5	Pubic region; penile shaft; upper limb; palm of the hand	Fever; headache; asthenia	No	No	CR
6	M	24	Caucasian (Italy)	MSM	Yes	No	Syphilis	Ulcerate lesion	1	Pubic region	Asthenia	Yes (inguinal area)	No	CR

Table S1: Clinical characteristics of patients with human monkeypox virus infection.

MSM: men who have sex with men; CR: complete remission.
